# Mitigating host microRNA interference to enhance mRNA vaccine efficacy in public health interventions

**DOI:** 10.1186/s40249-025-01308-6

**Published:** 2025-04-27

**Authors:** Tielong Xu, Ziqi Lin, Yicheng Yu, Muhammad Irfan, Munir Ahmed, Maya Septriana, Twinky Zebrina Cysta Sumantri, Anindini Winda Amalia, Bin Zheng

**Affiliations:** 1 Evidence-Based Medicine Research Center, Jiangxi University of Chinese Medicine, Nanchang, Jiangxi People’s Republic of China; 2 Affiliated hospital, Jiangxi University of Chinese Medicine, Nanchang, People’s Republic of China; 3 School of Pharmacy, Jiangxi University of Chinese Medicine, Nanchang, People’s Republic of China; 4 International Education College, Jiangxi University of Chinese Medicine, Nanchang, People’s Republic of China; 5 Department of Health, Faculty of Vocational Studies, Universitas Airlangga, Surabaya, Indonesia; 6 Department of Traditional Chinese Medicine, Faculty of Health, IIK Bhakti Wiyata Kediri, Kediri, Indonesia; 7 National Institute of Parasitic Diseases, Chinese Center for Disease Control and Prevention, and National Center for Tropical Diseases Research, Shanghai, People’s Republic of China

**Keywords:** MicroRNA, mRNA vaccine, mRNA vaccine technology, Host–pathogen interaction, Public health interventions

## Abstract

**Background:**

While mRNA vaccines represent a transformative platform for infectious disease control, their efficacy in antigen-presenting cells (APCs) remains vulnerable to endogenous regulatory networks, particularly microRNA (miR)-mediated translational suppression. This study addresses a critical gap in current vaccine design paradigms by systematically investigating host miR interference – an understudied barrier to robust antigen production.

**Main text:**

APCs express cell-type-specific miR repertoires capable of binding vaccine mRNAs through conserved seed sequences, as evidenced by synthesis of experimental data from 67 studies demonstrating miR-mediated repression of exogenous transcripts. To decode these inhibitory interactions, the commentary proposes an integrated multi-omics framework combining Argonaute immunoprecipitation with crosslinking-based miR-mRNA interactome sequencing, enabling precise mapping of miR-vaccine mRNA binding events in vaccine-transfected APCs. Furthermore, the commentary suggests two actionable strategies for evading miR interference: (1) Synonymous codon optimization at seed-match regions, achieving binding energy reduction while preserving antigenicity through degeneracy of genetic coding; (2) Targeted co-delivery of miR inhibitors. By bridging host RNA biology and vaccine engineering, this work provides a blueprint for developing miR-resistant mRNA vaccines for public health interventions.

**Conclusions:**

miRs may inhibit mRNA vaccine translation in APCs, potentially reducing antigen production and weakening the resulting immune response. To address this, next-generation mRNA vaccines should incorporate “miR-proofing” strategies during design to avoid miR interference.

**Graphical Abstract:**

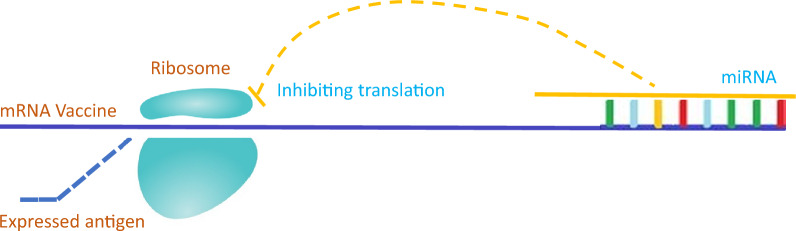

**Supplementary Information:**

The online version contains supplementary material available at 10.1186/s40249-025-01308-6.

## Background

The advent of messenger RNA (mRNA) vaccine technology has revolutionized infectious disease prevention through its rapid development cycle and unparalleled adaptability. Following six decades of foundational research—from mRNA discovery (1961) [1] to nucleoside modification breakthroughs [2]—this platform achieved global validation, with Pfizer-BioNTech vaccines demonstrating 95% efficacy and millions doses administered worldwide [3]. Accelerated by this success, mRNA vaccines are being actively developed against more than 17 priority infectious diseases, including malaria, tuberculosis, Zika, monkeypox, Lassa fever, chikungunya, and dengue fever [4]. Their significance in poverty-associated infectious diseases control stems from three unique advantages: (1) Cold-chain resilience—lipid nanoparticle (LNP) formulations now maintain stability for 4 weeks at 2–8 °C [5], critical for tropical regions; (2) Multiplexing capacity—single-dose combinations [4]; (3) Rapid response manufacturing [6].

mRNA acts as a “genetic courier”, carrying instructions from DNA in the nucleus to the cytoplasm’s “protein factories” (ribosomes). However, its path to medical relevance spanned six decades of breakthroughs and setbacks. Scientists first identified mRNA in 1961 [1], but like finding a lock without a key, it remained a biological curiosity for two decades, with no clear therapeutic applications. A pivotal moment arrived in 1984 [7] with the first laboratory synthesis of mRNA. This achievement unlocked the ability to design “custom genetic packages”—synthetic mRNAs that could theoretically instruct cells to produce any therapeutic protein [7]. Yet early forms faced three critical flaws, including fragility (degraded within minutes in the body), inflammatory triggers (mistaken by immune systems as viral invaders), and low yield (produced negligible amounts of protein) [2]. Groundbreaking work by Katalin Karikó and Drew Weissman (2000s) addressed these limitations [2], involving replacing uridine with pseudouridine (“molecular camouflage”) to evade immune detection and removing double-stranded RNA contaminants to reduce inflammation. These innovations culminated in Pfizer-BioNTech vaccines—the first FDA-approved mRNA therapeutics [8].

Despite monumental advances in nucleoside chemistry, the therapeutic efficacy of mRNA vaccines remains susceptible to interference from endogenous regulatory networks—particularly microRNAs (miRs). These small non-coding RNAs exhibit cell type-specific expression patterns and evolutionarily conserved seed sequence binding, enabling them to destabilize exogenous mRNA or repress translation via Argonaute (AGO)-mediated ribosome collision [9]. Whilst current design strategies prioritise optimisation of codon usage and evasion of innate immune sensors [2], they largely overlook miR-mRNA crosstalk. Critically, no systematic study has yet addressed the in vivo impact of miR activity on vaccine-derived antigen production. This commentary bridges this critical gap by adopting a three-phase conceptual framework. First, we introduce the fundamental duality of mRNA vaccine biology: while engineered mRNAs harness cellular translation machinery for antigen production, they simultaneously become substrates for endogenous miR regulatory networks. Second, through systematic synthesis of 67 experimental studies spanning dendritic cells (DCs), macrophages and B cells, we demonstrate that antigen-presenting cells (APCs)-specific miRs reduce model antigen expression via conserved seed sequence binding. Third, we propose to decipher miR-mRNA interaction dynamics using high-throughput sequencing of vaccine-transfected cells, and then to minimize miR-mediated inhibition by two orthogonal strategies, codon context refinement and co-delivery systems. This framework will advance the rational design of next-generation mRNA therapeutics with enhanced robustness against host miR interference.

## Main text

### Translation of mRNA vaccines in APCs is an initial step to elicit immunity

An mRNA vaccine delivers a genetic “instruction manual” (mRNA) that teaches cells to translate a harmless piece of the target pathogen (i.e., antigen). Specialized cells called APCs—the body’s “intelligence agents”—swallow the vaccine particles. These APCs perform three critical tasks: (1) capture: swallow the mRNA vaccine (endocytosis); (2) decode: release the mRNA into their “workspace” (cell cytoplasm); (3) produce: use ribosomes (cellular 3D printers) to build the antigen protein. The newly made antigen gets processed and displayed on the APC’s surface using the major histocompatibility complex (MHC) molecules—like holding up a “Wanted” poster to other immune cells [10]. Two MHC molecules exist, namely MHC I (by which the antigen is presented to killer T-cells) and MHC II (by which the antigen is presented to helper T-cells) [10]. The translated antigenic protein can stimulate the immune system in several ways, which have been detailed elsewhere [10]. The amount of antigen produced depends heavily on how efficiently the vaccine mRNA is translated in APCs. More antigen means stronger immune “training” before infection, longer-lasting protection hence better defense against real infections. From the process of immunization with mRNA vaccine, it can be seen that the translation of the antigen-encoding mRNA in APCs is a fundamental step in immunization, and the quantity of antigen protein produced can significantly impact the vaccine’s efficacy.

### miRs are universal inhibitors of mRNA translation

**Fig. 1:**
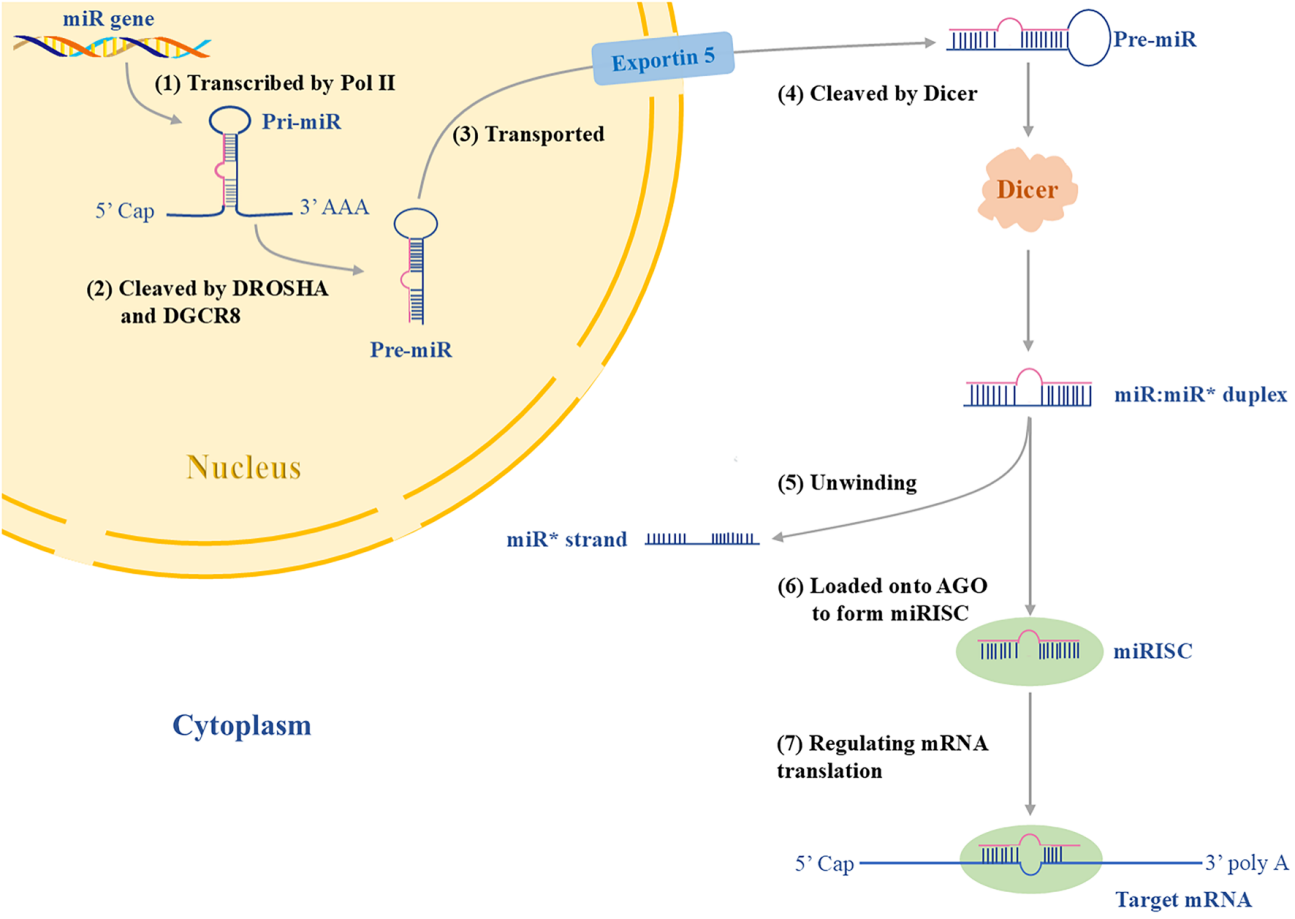
MicroRNA (miR) production pipeline: from DNA to gene silencing. The process includes seven key steps. (1) Transcription: RNA Polymerase II (Pol II) transcribes DNA into a long, folded RNA strand called pri-miR. (2) Nuclear Processing: In the nucleus, an enzyme team (DROSHA-DGCR8) cuts pri-miR into shorter precursor miR (pre-miR). (3) Export to Cytoplasm: Exportin-5 transports pre-miR to the cytoplasm (like a delivery truck moving goods to a factory floor). (4) Cytoplasmic Maturation: The enzyme Dicer slices pre-miR into a 22-nucleotide double-stranded RNA. (5) Duplex Dissociation: One strand becomes mature miR, while the other (miR*) usually gets degraded. (6) Loading onto AGO Protein: The mature miR associates with Argonaute proteins (AGO) to form the miR-induced silencing complex (miRISC) (assembling a "molecular search-and-silence squad"). (7) Target Recognition and Gene Regulation: The miRISC scans mRNAs for matching sequences. Once found, it either mutes translation or destroys mRNA. [9]

miRs are tiny RNA molecules (about 22 nucleotides long) that act as universal “protein production controllers”. Unlike traditional genes, they don’t code for proteins themselves—instead, they fine-tune how much protein other genes make [9]. miRs are first transcribed in the nucleus as long “primary miR strands” (pri-miRs), which are then trimmed into shorter, functional forms. These mature miRs team up with a protein complex called miR-induced silencing complex (miRISC) (like a “molecular search-and-silence squad”). The miRISC scans mRNAs in the cell, looking for matching genetic sequences. When they find a match, they either cut the mRNA or block translation [9]. miRs aren’t confined to their home cell. They can travel between cells inside tiny “biological packages” (exosomes) or hitch rides on proteins. Once delivered, they regulate genes in distant cells, much like text messages sent between phones [11]. In these ways, miRs are involved in a wide range of biological and pathological processes, including cell proliferation, differentiation, apoptosis, tumorigenesis, and viral infection [11]. Figure 1 illustrates the intricate process of miR biosynthesis, including the steps from pri-miR transcription to the formation of the mature miR, as well as the subsequent functions of miRs in post-transcriptional gene regulation.

### miRs may inhibit the translation of mRNA vaccine in APCs

Despite the global success of mRNA vaccines, scientists have yet to unravel whether miRs act as “molecular brakes”, quietly reducing vaccine efficiency in key immune cells, i.e., APCs. Since vaccine mRNA follows the same cellular pathways as regular mRNA, this review investigates miR-mediated suppression of standard mRNAs in APCs to predict their potential impact on vaccines.

DCs, macrophages, and B cells are commonly identified as the main types of APCs [12]. DCs are the immune system’s “scouts” that first detect invaders. Macrophages can be thought like “Cleanup crews” that engulf and destroy pathogens. B cells are “Antibody factories” that produce targeted defenses [12]. Appendix Table 1 synthesizes experimental evidence from recent studies demonstrating miR-mediated translational repression of mRNAs in three major APC types. The table catalogues APC-specific miRs (e.g., miR-150-5p [13], miR-155 [14]) and their corresponding target mRNAs, highlighting sequence-specific interactions that suppress antigen production. Notably, each APC type harbors miRs that “stick to” specific mRNAs. These miRs act as “patrol units”, either blocking protein production or shredding mRNA.

**Fig. 2:**
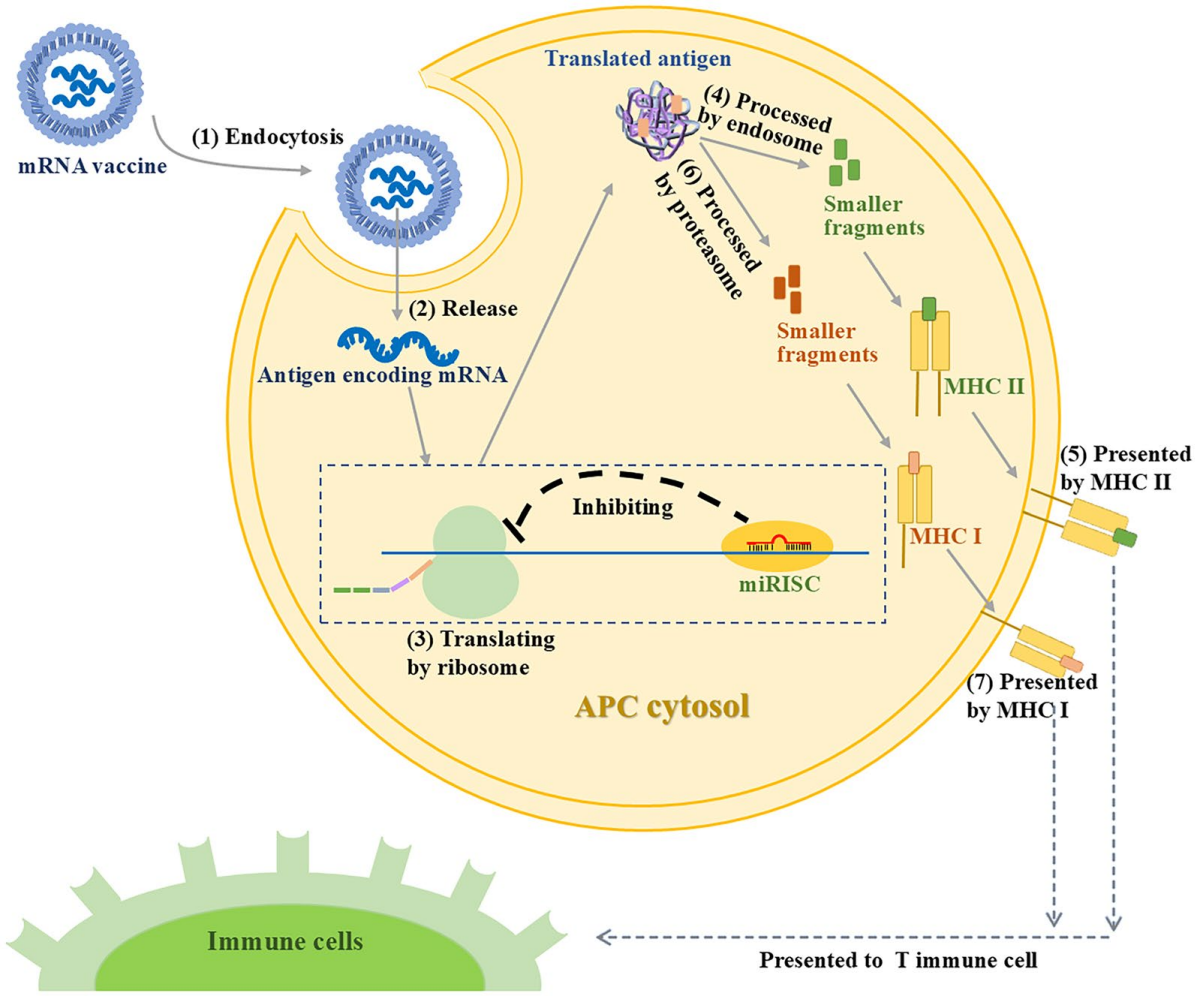
MicroRNAs (miRs) have been proposed to suppress the translation of mRNA vaccine within antigen-presenting cells (APCs). This figure illustrates how miRs could disrupt mRNA vaccine effectiveness in APCs, reducing immune protection. Within these cells, the antigen-encoding mRNA from the mRNA vaccine is released and translated into proteins. These proteins are then processed and presented in two distinct pathways to induce a specific immune response, as outlined by steps (1), (2), (3), (4), (5), or alternatively, (1), (2), (3), (6), (7) [10]. However, miRs can bind to complementary sequences on the mRNA molecules, forming complexes with proteins such as Argonaute proteins, collectively known as the miR-induced silencing complex (miRISC). This interaction leads to translational repression or degradation of the mRNA, thereby inhibiting the production of antigens derived from the vaccine, as indicated by the content within the dashed line. Consequently, this interference could diminish both the magnitude and efficacy of the immune response elicited

These findings demonstrate that miRs universally inhibit mRNA translation across APCs. Such suppression suggests that miRs may directly reduce antigen yields by targeting vaccine-encoding mRNAs, thereby compromising immune efficacy (Fig. 2). As a result, it is imperative to consider the inhibitory effects of miRs during the development of mRNA vaccines, especially the miRs which are highly expressed in APCs, such as miR-150-5p [13], miR-155 [14], and so on.

### Method to identify the specific miRs that inhibit the translation of mRNA vaccines within APCs

**Fig. 3:**
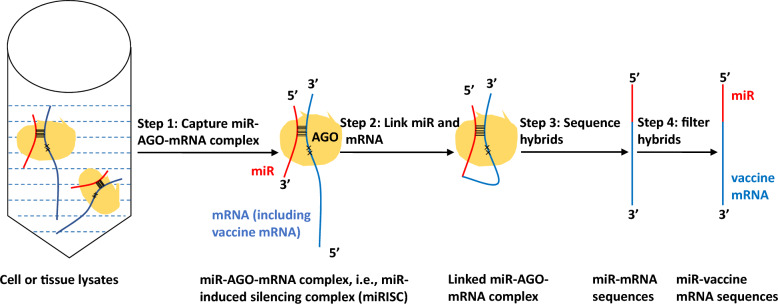
Schematic of the principles and procedures for detecting the miR-mRNA interactome to identify miRs that can bind to the vaccine mRNA. First, antigen-presenting cells (APCs) internalizing the mRNA vaccine are lysed to release intracellular components, miR-AGO-mRNA complexs. The miR-AGO-mRNA complexes are selectively captured using Argonaute (AGO)-specific antibody immunoprecipitation, labeled as “Step 1: Capture miR-AGO-mRNA Complex” to emphasize this critical isolation step. Next, an RNA ligase enzyme facilitates intermolecular ligation, covalently linking the 3’ end of the miR to the 5’ end of the vaccine mRNA strand (labeled as “Step 2: Link miR and mRNA”). Color-coding is applied to distinguish molecular components: miR (red), vaccine mRNA (blue), AGO protein (yellow). Finally, the chimeric miR-mRNA hybrids are purified and subjected to high-throughput sequencing (labeled as “Step 3: Sequence hybrids”). Furthermore, miR-mRNA hybrids with vaccine mRNA at the 3’ end are selected out by searching against the vaccine mRNA sequences, hence the miRs that can bind to the vaccine mRNA are obtained (labeled as “Step 4: Filter hybrids”). [15–18]

The regulatory role of miRs in mRNA translation is always inhibitory [9]. To mitigate the inhibitory effects of miRs on mRNA vaccine translation, it is crucial to identify the miRs capable of binding to the antigen-encoding mRNA within APCs. During the process of miR regulation on mRNAs, miR, mRNA, and AGO proteins are bound together, forming what is termed the “miR-AGO-mRNA complex”. Consequently, investigators have developed a protocol to delineate the miR-mRNA interactome by focusing on the “miR-AGO-mRNA complex”, as outlined below [15–18]:

1. Use AGO immunoprecipitation (a method like magnetic fishing rods) to capture all “miR-AGO-mRNA complex” from cells—the “smoking gun” evidence of miRs activities.
2. Apply ligase enzymes (molecular glue) to permanently link the 3’ ends of miRs to the 5’ ends of their target mRNAs, creating miR-mRNA hybrids—like handcuffing a thief to their loot.
3. Sequence these hybrids to reveal exact miR-mRNA pairs—think of it as scanning a criminal database to match fingerprints.
4. Use bioinformatics to identify which miRs target vaccine mRNA. This generates a “most wanted” list of interfering miRs. (Fig. 3)

The miR-mRNA interactome sequencing technology has been effectively utilized to profile the miRs targeting particular mRNAs, such as Hepatitis C virus mRNA [15], nonmetastatic cells 1 (NME1) [16], AT-rich interacting domain-containing protein 1A (ARID1A) [17], and Epstein Barr virus mRNA [18]. Initially designed for the analysis of tissue samples, miR-mRNA interactome sequencing technology is now capable of being applied to samples containing 10 million cells or fewer [15–18]. Consequently, it can be inferred that this technology is suitable for detecting miRs capable of binding to antigen-encoding mRNAs within APCs. APCs, which have been invaded by a mRNA vaccine either in vitro or in vivo, can serve as the subjects for detection (Fig. 3). Detailed procedures for detecting the miR-mRNA interactome have been provided as in Appendix Fig. 1.

### Strategies to mitigate the inhibitory impact of miRs on mRNA vaccine translation in APCs

When a miR is found to bind to an mRNA vaccine within APCs, the key challenge is how to block its ability to suppress vaccine mRNA translation. We propose two strategies to overcome this issue. The first approach involves redesigning the vaccine mRNA’s genetic code. miRs recognize their targets using a short “molecular GPS” called the seed sequence (positions 2–8 at the miR’s 5’ end) [9]. While the miR’s 3’ end can sometimes help strengthen binding, the seed sequence is the primary driver of this interaction [9]. By introducing tiny changes—even altering a single genetic “letter” (point mutations)—in the vaccine mRNA’s seed-binding region, researchers can disrupt miR binding [19]. Crucially, these changes must not alter the protein the mRNA encodes. This is possible due to the genetic code’s built-in redundancy: most amino acids can be spelled by multiple three-letter combinations (codons) [20]. For example, the amino acid arginine can be encoded by CGA, CGT, or other codons. By swapping synonymous codons (e.g., changing CGA to CGT), scientists can tweak the mRNA sequence to evade miRs while preserving the vaccine’s protein product [19, 20].

The second strategy focuses on silencing harmful miRs directly. Advances in drug delivery now allow miR inhibitors—molecules that neutralize specific miRs—to be precisely delivered to APCs in living organisms [21, 22]. For instance, in patients with viral pneumonia, overactive miR-21 in lung macrophages exacerbates inflammation and scarring. To combat this, researchers attached a miR-21 inhibitor to trimannose, a sugar molecule naturally recognized by lung macrophages. When inhaled by mice, this inhibitor rapidly reached its target, reducing inflammation, preventing lung damage, and restoring normal macrophage function [21].

## Discussion

Compared to traditional protein-based medicines, mRNA therapies hold unique advantages. They are faster and cheaper to produce, can be easily mixed into combination vaccines [4, 6]. Scientists are developing multi-target vaccines and expanding beyond infectious diseases to tackle cancer and autoimmune disorders [4]. However, there’s a catch: miRs—tiny RNA molecules that naturally act as “cellular brakes”—may sabotage mRNA vaccines by blocking their protein production. This interference risks reducing the vaccine’s effectiveness, much like static noise disrupting a radio signal. Our work found a “miR-proofing” strategy to identify and neutralize miR interference, enhancing mRNA vaccine functionality. This framework provides both mechanistic insights into host–pathogen molecular conflicts and actionable solutions for developing miR-resistant mRNA vaccines. In our opinion, eliminating miR-mediated suppression effect on vaccine mRNA could reduce required doses, enabling cost-effective coverage for at-risk populations lacking routine immunization access. This is particularly crucial for resource-limited regions (e.g., developing world) where dose optimization directly impacts immunization performance and incidence rates of infectious diseases.

However, implementing this strategy may be faced with some technical challenges, such as codon optimization complexity and off-target effects. Synonymous codon substitutions, though promising for evading miR binding, may inadvertently reduce vaccine efficacy due to cell-specific codon preferences in APCs or unintended structural alterations in the mRNA [20]. Additionally, achieving precise delivery of miR inhibitors to all relevant APCs remains challenging, with off-target delivery risking unintended biological consequences. Advancing beyond current limitations will require next-generation machine learning platforms to optimize codon selection and tissue-specific delivery systems that balance efficacy with safety.

## Conclusions

miRs may inhibit mRNA vaccine translation in APCs, potentially reducing antigen production and weakening the resulting immune response. To address this, next-generation mRNA vaccines should incorporate “miR-proofing” strategies during design to avoid miR interference.

## Supplementary Information

Additional file 1. **Figure 1** Detailed procedures for detecting the miR-mRNA interactome to identify miRs that can bind to the vaccine mRNA.

Additional file 2. **Table 1** Evidence of miR-Mediated inhibition of mRNA translation in APCs

## Data Availability

All data supporting the findings of this study are included in the article.

## Abbreviations

MHC: Histocompatibility complex
Pri-miR: Primary microRNAs
Pol II: RNA polymerase II
Pre-miR: Precursor microRNA
DROSHA: Drosha ribonuclease III
DGCR8: DiGeorge syndrome critical region 8
AGO: Argonaute proteins
miRISCs: MicroRNA-induced silencing complexes
APCs: Antigen-presenting cells.
DC: Dendritic cells
miR: MicroRNA
ARID1A: AT-rich interacting domain-containing protein 1A
NME1: Nonmetastatic cells 1
