# Supplementary material for: Mitigating host microRNA interference to enhance mRNA vaccine efficacy in public health interventions
Source: Infect Dis Poverty. 2025 Apr 27;14:32. doi: 10.1186/s40249-025-01308-6 (PMC12034212; doi:10.1186/s40249-025-01308-6)
Supplement: Supplementary file 1 — Additional file 1. Figure 1 Detailed procedures for detecting the miR-mRNA interactome to identify miRs that can bind to the vaccine mRNA. [file 40249_2025_1308_MOESM1_ESM.docx]

**254nm** **ultraviolet (UV)**

**①UV Crosslinking:** Expose samples to 254 nm ultraviolet (UV) light to stabilize the “miRNA-AGO-target mRNA” silencing complex.

**②Cell Lysis and DNA Digestion:** Lyse tissue cells and digest residual DNA using DNase.

**③RNA Fragmentation:** Treat samples with RNase to cleave target mRNA into appropriately sized fragments.

**④AGO Immunoprecipitation:** Use an AGO-specific antibody to immunoprecipitate the AGO protein (antigen), thereby isolating the "miRNA-AGO-target mRNA" complex from the lysate.

**⑤Washing:** Perform thorough washing steps to remove nonspecific bindings.

**※Antigen-presenting cells (APCs) internalizing the mRNA vaccine**


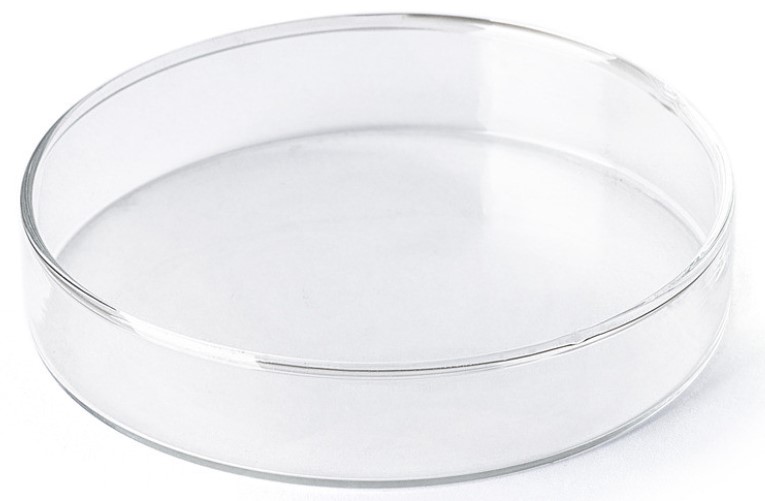


3’P

**Target mRNA**

**miR**

5’P

**AGO Protein**

**※The miR-AGO-mRNA complex was captured via antigen-antibody binding (immunoprecipitation)**

3’OH

**⑥5’ Phosphorylation of Target mRNA:** Phosphorylate the 5’ end of the target mRNA to enable ligation to the miRNA 3’ end.

**⑦Ligation with T4 RNA Ligase:** Use T4 RNA ligase to covalently link the 5’ end of the target mRNA to the 3’ end of the miRNA, generating miRNA-target mRNA hybrid strands.

**⑧3’ Dephosphorylation and Linker Addition:** Dephosphorylate the 3’ end of the hybrid strands and ligate a 3’ sequencing linker.

5’OH

3’P

5’P

**※Linked miR-AGO-mRNA complex**

**⑨RNA Extraction:** Purify the RNA hybrid strands

**⑩5’ Sequencing Linker Ligation:** Attach a 5’ sequencing linker to the hybrid strands.

⑪**Library Preparation and Sequencing:** Perform PCR amplification to construct the sequencing library, followed by high-throughput sequencing.

**※miR-mRNA sequences**

5’

3’

⑫**Hybrids filter**: Hybrid strands with vaccine mRNA at the 3’ end were analyzed by searching against the vaccine mRNA sequences.

**※miR-vaccine mRNA sequences**

3’

5’

**⑬Analysis:** Process sequencing data to identify miRNA-vaccine mRNA interactions and validate targeting specificity.

**miR profile**

**Vaccine mRNA**

**※miR profile that can bind to the vaccine mRNA**

**Appendix Figure 1. Schematic of the principles and procedures for detecting the miR-mRNA interactome to identify miRs that can bind to the vaccine mRNA.** MicroRNAs (miRs) regulate gene expression post-transcriptionally by forming the “miR-AGO-target mRNA complex” (termed miRISC). However, during immunoprecipitation and purification procedures, the target mRNA within this complex is prone to detachment. To address this, UV crosslinking (indicated by "×" in the figure) can be applied to stabilize the interaction between AGO and the target mRNA, thereby forming a robust “miR-AGO-target mRNA complex” resistant to experimental washing steps. Following immunoprecipitation of the “miR-AGO-target mRNA complex”, the 3’ end of the miR and the 5’ end of the mRNA are chemically modified. T4 RNA ligase is then used to covalently ligate the miR’s 3’ end to the mRNA’s 5’ end, enabling precise capture of functional miRNAs and their corresponding target mRNAs.
