# Supplementary material for: Mitigating host microRNA interference to enhance mRNA vaccine efficacy in public health interventions
Source: Infect Dis Poverty. 2025 Apr 27;14:32. doi: 10.1186/s40249-025-01308-6 (PMC12034212; doi:10.1186/s40249-025-01308-6)
Supplement: Supplementary file 2 — Additional file 2. Table 1 Evidence of miR-Mediated inhibition of mRNA translation in APCs [file 40249_2025_1308_MOESM2_ESM.docx]

Appendix Table 1 Evidence of miR-Mediated inhibition of mRNA translation in APCs

| **miRs** | **Inhibited**  **mRNA** | **miRs** | **Inhibited mRNA** | **miRs** | | **Inhibited**  **mRNA** | |
| --- | --- | --- | --- | --- | --- | --- | --- |
| In dendritic cells | |  | |  |  | |  |
| miR-155 | C/EBP [1] | miR-133b | Zbtb7b [2] | miR-144 | | IRF5 [3] | |
|  | Arg2 [4] | miR-133a | RBP-J [5] | miR-150-5p | | TREM-1 [6] | |
|  | ENTPD [7] | miR-148a | DNMT 1 [8] | miR-146 | | IRAKI [9] | |
|  | Ship1 [10] |  | MAFB [11] | miR-23b | | p50/p65 [12] | |
|  | IL‐17 [13] | miR-650 | MXA [14] | miR-4448 | | p53 [15] | |
| miR-9 | REST/NRSF [16] | let-7i | IL-10 [17] | miR‐142 | | IL‐1b, SOCS1 [13] | |
|  | Pcgf6 [18] |  | TGF‐β [13] | miR-194-5p | | B7 homolog 1 [19] | |
| miR-100-5p | TLR4 [20] | miR-34 | AXL [21] | miR-5119 | | PD-L1, IDO 2 [22] | |
| miR-214 | β-catenin [23] |  | Jagged-1 [24] | miR-21 | | PTEN [25] | |
| miR-22 | p38 [26] | miR-181-5p | TNF-α [27] |  | | WNT1 [24] | |
| miR-106b | EGR-2 [28] | miR-31 | IL-4 [29] | miR-155 | | IFN-γ [13] | |
| miR-106a | STAT3 [30] | miR-223-3p | NLRP3 [31] | …… | | …… | |
| In macrophages | |  |  |  | |  | |
| miR-155 | C/EBP [1] | miR-126 | MEKK2 [32] | miR-183-5p | | FoxO1 [33] | |
|  | SOCS1 [34] | let-7c | IL-6 [35] | miR-29a | | PIK3CA [36] | |
| miR-202-5p | Bcl-2 [37] | miR-4766 | VEGFA [38] | miR-224 | | WNT 9a [39] | |
| miR-146b | IRF5 [40] | miR-382 | SIRP-α [41] | miR-125b-5p | | A20 [42] | |
| miR-146a | IRAK-1 [43] | miR-30d-5p | HDAC9 [44] | miR-22-3p | | PER2 [45] | |
| miR-146-5p | TRAF6 [46] | miR-30b | MALAT [47] | miR-660 | | KLHL21 [48] | |
| miR-21a-5p | GATA2 [49] | miR-27a-3p | EZH1 [50] | miR-32 | | PTEN [51] | |
| miR-139-3p | STAT 1 [52] | miR-1224 | MSI2 [53] | miR-214-3p | | GSK3B [54] | |
| miR-342-3p | Metadherin [55] | miR-26a | ATF2 [56] | …… | | …… | |
| In B cells |  |  |  |  | |  | |
| miR-107 | ATG12 [57] | miR-155 | SMAD5 [58] | miR-150 | | c-Myb [59] | |
| miR-30b-5p | BAFF [60] | miR-146a | FAS [61] |  | | FLT3 [62] | |
| miR-320a | MMP-9 [63] | miR-17~92 | BIM [64] | miR-34 | | Foxp 1 [65] | |
| miR-181b | AID [66] |  | PTEN [67] | …… | | …… | |

Note: “……” indicates that there is a wealth of additional evidence suggesting that miRs inhibit mRNA translation in APCs.

The abbreviations presented in the table are correspondingly defined as follows.

APCs: Antigen-presenting cells; AID: Activation-induced cytidine deaminase; Arg2: Arginases 2; ATF2: Transcription factor 2; ATG12: Autophagy-related protein 12; AXL: Axl receptor tyrosine kinase; BAFF: B-cell activating factor; Bcl-2: B-cell leukemia/lymphoma 2; BIM: Bcl-2 Interacting Mediator of Cell Death; C/EBP beta: Transcription factor C/EBP beta; c-Myb: v-Myb myeloblastosis viral oncogene homolog; CNS: central nervous system; DC: dendritic cells; DNMT1: DNA methyltransferase 1; Egr2: Early growth response 2; ENTPD: Ectonucleoside triphosphate diphosphohydrolases; EZH1: Enhancer of zeste homologue 1; FAS: Surface death receptor; FLT3: FMS-like tyrosine kinase 3; FoxO1: Forkhead box protein O1; Foxp1: Forkhead box transcription factor 1; FLT3: Fms Related Receptor Tyrosine Kinase 3; GATA2: Zinc finger transcription factors GATA-binding factor 2; GSK3B: Glycogen synthase kinase 3 beta; HDAC9: Deacetylase 9; IDO2: indoleamine-2,3-dioxygenase 2; IFN: interferon; IFN-γ: Interferon‐γ; IL-10: Interleukin‐10; IL-17: Interleukin‐17; IL-1b: Interleukin‐1b; IL-4: Interleukin‐4; IL-6: interleukin-6; IRAK-1: interleukin-1 receptor-associated kinase 1; IRAKI: Receptor-associated Kinases I; IRF5: Interferon regulatory factor 5; KLHL21: Kelch-like Protein 21; MAFB: v-maf avian musculoaponeurotic fibrosarcoma oncogene homolog B; MALAT: metastasis-associated lung adenocarcinoma transcript; MAPKs: mitogen-activated protein kinases signaling STAT3; MEKK2: Mitogen-Activated Protein Kinase Kinase (MEK) and Extracellular Signal-Regulated Kinase (ERK) pathway kinase 2; MMP-9: Matrix metallopeptidase-9; MSI2: musashi RNA binding protein 2; MTDH: Metadherin; MXA: well-known interferon-stimulated genes; MYSM1: MYSM1 Myb like, SWIRM and MPN domains 1; NF-Кb: nuclear factor kappa signaling; NKT: natural killer T; NLRP3: NLR family protein containing a pyrin domain 3; NKT17: natural killer T cell; Pcgf6: Transcriptional repressor Polycomb group factor 6; PD-L1: Programmed death-ligand 1; PER2: Period circadian protein 2; PI3K/AKT: Phosphatidylinositol 3-kinase / Protein kinase B; PIK3CA: Phosphoinositide 3-kinase catalytic subunit alpha; PTEN: Phosphatase and tensin homolog; RBP-J: Recombination signal binding protein for immunoglobulin kappa J region; REST/NRSF: Global transcriptional repressor element 1 silencing transcription factor; SHIP1: Lipid phosphatases, the 5’-inositol phosphatase 1; SIRP-α: Signal regulatory protein alpha; SMAD5: Homologs of the Drosophila melanogaster protein mothers against decapentaplegic and C. elegans small body size 5; SOCS1: Suppressor of cytokine signaling 1; STAT1: Signal transducer and activator of transcription 1; STAT3: signal transducerand activator of transcription-3; TGF-β: Transforming Growth Factor Beta; Th2: type 2 helper T; TLR4: Toll-like receptor 4; TNF-α: Tumor Necrosis Factor Alpha; TRAF-6: Tumor necrosis factor receptor-associated factor 6; Treg: regulatory T; TREM-1: Triggering receptors expressed on myeloid cells-1; VEGFA: Vascular Endothelial Growth Factor A; WNT 9a: WNT 9a; WNT1: WNT family member 1; Zbtb7b: Zinc finger and BTB domain-containing protein 7B; β-catenin: β-catenin; EGR-2: Early growth response 2.

1. Dueck A, Eichner A, Sixt M, Meister G. A miR-155-dependent microRNA hierarchy in dendritic cell maturation and macrophage activation. FEBS Lett. 2014 Feb 14;588(4):632-40.

2. Di Pietro C, De Giorgi L, Cosorich I, Sorini C, Fedeli M, Falcone M. MicroRNA-133b Regulation of Th-POK Expression and Dendritic Cell Signals Affect NKT17 Cell Differentiation in the Thymus. J Immunol. 2016 Oct 15;197(8):3271-80.

3. Lin Z, Xie X, Gu M, Chen Q, Lu G, Jia X, et al. microRNA-144/451 decreases dendritic cell bioactivity via targeting interferon-regulatory factor 5 to limit DSS-induced colitis. Front Immunol. 2022;13:928593.

4. Dunand-Sauthier I, Irla M, Carnesecchi S, Seguin-Estevez Q, Vejnar CE, Zdobnov EM, et al. Repression of arginase-2 expression in dendritic cells by microRNA-155 is critical for promoting T cell proliferation. J Immunol. 2014 Aug 15;193(4):1690-700.

5. Gao X, Han D, Fan W. Down-regulation of RBP-J mediated by microRNA-133a suppresses dendritic cells and functions as a potential tumor suppressor in osteosarcoma. Exp Cell Res. 2016 Dec 10;349(2):264-72.

6. Yue C, Wang W, Gao S, Ye J, Zhang T, Xing Z, et al. Agomir miRNA-150-5p alleviates pristane-induced lupus by suppressing myeloid dendritic cells activation and inflammation via TREM-1 axis. Inflamm Res. 2023 Jul;72(7):1391-408.

7. Zech A, Ayata CK, Pankratz F, Meyer A, Baudiss K, Cicko S, et al. MicroRNA-155 modulates P2R signaling and Th2 priming of dendritic cells during allergic airway inflammation in mice. Allergy. 2015 Sep;70(9):1121-9.

8. Liu L, Yi H, Wang C, He H, Li P, Pan H, et al. Integrated Nanovaccine with MicroRNA-148a Inhibition Reprograms Tumor-Associated Dendritic Cells by Modulating miR-148a/DNMT1/SOCS1 Axis. J Immunol. 2016 Aug 15;197(4):1231-41.

9. Park H, Huang X, Lu C, Cairo MS, Zhou X. MicroRNA-146a and microRNA-146b regulate human dendritic cell apoptosis and cytokine production by targeting TRAF6 and IRAK1 proteins. J Biol Chem. 2015 Jan 30;290(5):2831-41.

10. Yan S, Yim LY, Tam RC, Chan A, Lu L, Lau CS, et al. MicroRNA-155 Mediates Augmented CD40 Expression in Bone Marrow Derived Plasmacytoid Dendritic Cells in Symptomatic Lupus-Prone NZB/W F1 Mice. Int J Mol Sci. 2016 Aug 6;17(8).

11. Meng Y, Li J, Ye Z, Yin Z, Sun Q, Liao Z, et al. MicroRNA-148a facilitates inflammatory dendritic cell differentiation and autoimmunity by targeting MAFB. JCI Insight. 2020 Apr 23;5(8).

12. Wu J, Ji C, Cao F, Lui H, Xia B, Wang L. Bone marrow mesenchymal stem cells inhibit dendritic cells differentiation and maturation by microRNA-23b. Biosci Rep. 2017 Apr 30;37(2).

13. Taghikhani A, Hassan ZM, Ebrahimi M, Moazzeni SM. microRNA modified tumor-derived exosomes as novel tools for maturation of dendritic cells. J Cell Physiol. 2019 Jun;234(6):9417-27.

14. Pichulik T, Khatamzas E, Liu X, Brain O, Delmiro Garcia M, Leslie A, et al. Pattern recognition receptor mediated downregulation of microRNA-650 fine-tunes MxA expression in dendritic cells infected with influenza A virus. Eur J Immunol. 2016 Jan;46(1):167-77.

15. Banos-Lara MDR, Zabaleta J, Garai J, Baddoo M, Guerrero-Plata A. Comparative analysis of miRNA profile in human dendritic cells infected with respiratory syncytial virus and human metapneumovirus. BMC Res Notes. 2018 Jul 3;11(1):432.

16. Giusti SA, Vogl AM, Brockmann MM, Vercelli CA, Rein ML, Trumbach D, et al. MicroRNA-9 controls dendritic development by targeting REST. Elife. 2014 Nov 18;3.

17. Sun Y, Jin X, Liu X, Zhang M, Liu W, Li Z, et al. MicroRNA let-7i regulates dendritic cells maturation targeting interleukin-10 via the Janus kinase 1-signal transducer and activator of transcription 3 signal pathway subsequently induces prolonged cardiac allograft survival in rats. J Heart Lung Transplant. 2016 Mar;35(3):378-88.

18. Cordeiro B, Jeon P, Boukhaled GM, Corrado M, Lapohos O, Roy DG, et al. MicroRNA-9 Fine-Tunes Dendritic Cell Function by Suppressing Negative Regulators in a Cell-Type-Specific Manner. Cell Rep. 2020 May 5;31(5):107585.

19. Wang Y, Petrikova E, Gross W, Sticht C, Gretz N, Herr I, et al. Sulforaphane Promotes Dendritic Cell Stimulatory Capacity Through Modulation of Regulatory Molecules, JAK/STAT3- and MicroRNA-Signaling. Front Immunol. 2020;11:589818.

20. Aungier SR, Ohmori H, Clinton M, Mabbott NA. MicroRNA-100-5p indirectly modulates the expression of Il6, Ptgs1/2 and Tlr4 mRNA in the mouse follicular dendritic cell-like cell line, FL-Y. Immunology. 2015 Jan;144(1):34-44.

21. Kurowska-Stolarska M, Alivernini S, Melchor EG, Elmesmari A, Tolusso B, Tange C, et al. MicroRNA-34a dependent regulation of AXL controls the activation of dendritic cells in inflammatory arthritis. Nat Commun. 2017 Jun 22;8:15877.

22. Zhang M, Shi Y, Zhang Y, Wang Y, Alotaibi F, Qiu L, et al. miRNA-5119 regulates immune checkpoints in dendritic cells to enhance breast cancer immunotherapy. Cancer Immunol Immunother. 2020 Jun;69(6):951-67.

23. Gu C, Zhou XD, Yuan Y, Miao XH, Liu Y, Ru YW, et al. MicroRNA-214 induces dendritic cell switching from tolerance to immunity by targeting beta-Catenin signaling. Int J Clin Exp Pathol. 2015;8(9):10050-60.

24. Hashimi ST, Fulcher JA, Chang MH, Gov L, Wang S, Lee B. MicroRNA profiling identifies miR-34a and miR-21 and their target genes JAG1 and WNT1 in the coordinate regulation of dendritic cell differentiation. Blood. 2009 Jul 9;114(2):404-14.

25. Liu F, Liu C, Hu X, Shang Y, Wu L. MicroRNA-21: A Positive Regulator for Optimal Production of Type I and Type III Interferon by Plasmacytoid Dendritic Cells. Front Immunol. 2017;8:947.

26. Liang X, Liu Y, Mei S, Zhang M, Xin J, Zhang Y, et al. MicroRNA-22 impairs anti-tumor ability of dendritic cells by targeting p38. PLoS One. 2015;10(3):e0121510.

27. Zhu J, Wang FL, Wang HB, Dong N, Zhu XM, Wu Y, et al. TNF-alpha mRNA is negatively regulated by microRNA-181a-5p in maturation of dendritic cells induced by high mobility group box-1 protein. Sci Rep. 2017 Sep 25;7(1):12239.

28. Tang H, Jiang H, Zheng J, Li J, Wei Y, Xu G, et al. MicroRNA-106b regulates pro-allergic properties of dendritic cells and Th2 polarisation by targeting early growth response-2 in vitro. Int Immunopharmacol. 2015 Oct;28(2):866-74.

29. Hoye ML, Archambault AS, Gordon TM, Oetjen LK, Cain MD, Klein RS, et al. MicroRNA signature of central nervous system-infiltrating dendritic cells in an animal model of multiple sclerosis. Immunology. 2018 Sep;155(1):112-22.

30. Zhou H, Sun C, Li C, Hua S, Li F, Li R, et al. The MicroRNA-106a/20b Strongly Enhances the Antitumour Immune Responses of Dendritic Cells Pulsed with Glioma Stem Cells by Targeting STAT3. J Immunol Res. 2022;2022:9721028.

31. Chen L, Hou X, Zhang M, Zheng Y, Zheng X, Yang Q, et al. MicroRNA-223-3p modulates dendritic cell function and ameliorates experimental autoimmune myocarditis by targeting the NLRP3 inflammasome. Mol Immunol. 2020 Jan;117:73-83.

32. Li J, Liu Y, Lai W, Song L, Deng J, Li C, et al. MicroRNA-126 regulates macrophage polarization to prevent the resorption of alveolar bone in diabetic periodontitis. Arch Oral Biol. 2023 Jun;150:105686.

33. Tang DS, Cao F, Yan CS, Cui JT, Guo XY, Cheng L, et al. Acinar Cell-Derived Extracellular Vesicle MiRNA-183-5p Aggravates Acute Pancreatitis by Promoting M1 Macrophage Polarization Through Downregulation of FoxO1. Front Immunol. 2022;13:869207.

34. Chang Y, Chen X, Tian Y, Gao X, Liu Z, Dong X, et al. Downregulation of microRNA-155-5p prevents immune thrombocytopenia by promoting macrophage M2 polarization via the SOCS1-dependent PD1/PDL1 pathway. Life Sci. 2020 Sep 15;257:118057.

35. Liu T, Zhang Z, Shen W, Wu Y, Bian T. MicroRNA Let-7 Induces M2 Macrophage Polarization in COPD Emphysema Through the IL-6/STAT3 Pathway. Int J Chron Obstruct Pulmon Dis. 2023;18:575-91.

36. Shao W, Wang S, Wang X, Yao L, Yuan X, Huang D, et al. miRNA-29a inhibits atherosclerotic plaque formation by mediating macrophage autophagy via PI3K/AKT/mTOR pathway. Aging (Albany NY). 2022 Mar 14;14(5):2418-31.

37. Xu F, Yao F, Ning Y. MicroRNA-202-5p-dependent inhibition of Bcl-2 contributes to macrophage apoptosis and atherosclerotic plaque formation. Gene. 2023 May 30;867:147366.

38. Chen C, Huang Z, Tan X, Wang R, Liu J, Zhang M. The microRNA-4766/VEGFA axis mediates macrophage M2-type polarization to inhibit colorectal cancer proliferation and migration. Pathol Res Pract. 2023 Oct;250:154767.

39. Luo Z, Qi B, Sun Y, Chen Y, Lin J, Qin H, et al. Engineering Bioactive M2 Macrophage-Polarized, Anti-inflammatory, miRNA-Based Liposomes for Functional Muscle Repair: From Exosomal Mechanisms to Biomaterials. Small. 2022 Aug;18(34):e2201957.

40. Wang C, Cheng H, Yan F, Zhang H, Zhang J, Li C, et al. MicroRNA-146b protects kidney injury during urinary tract infections by modulating macrophage polarization. mBio. 2023 Nov 1:e0209423.

41. Wang X, Jia P, Ren T, Zou Z, Xu S, Zhang Y, et al. MicroRNA-382 Promotes M2-Like Macrophage via the SIRP-alpha/STAT3 Signaling Pathway in Aristolochic Acid-Induced Renal Fibrosis. Front Immunol. 2022;13:864984.

42. Luo XB, Li LT, Xi JC, Liu HT, Liu Z, Yu L, et al. Negative pressure promotes macrophage M1 polarization after Mycobacterium tuberculosis infection via the lncRNA XIST/microRNA-125b-5p/A20/NF-kappaB axis. Ann N Y Acad Sci. 2022 Aug;1514(1):116-31.

43. Yan Z, Wen JX, Cao XS, Zhao W, Han YL, Wen XH, et al. Tumor cell-derived exosomal microRNA-146a promotes non-small cell lung cancer cell invasion and proliferation by inhibiting M1 macrophage polarization. Ann Transl Med. 2022 Dec;10(24):1307.

44. Bai K, Li J, Lin L, Zhang Q, Zhong J, Liu X, et al. Placenta exosomal miRNA-30d-5p facilitates decidual macrophage polarization by targeting HDAC9. J Leukoc Biol. 2023 May 2;113(5):434-44.

45. Liu C, Liang T, Zhang Z, Chen J, Xue J, Zhan X, et al. Transfer of microRNA-22-3p by M2 macrophage-derived extracellular vesicles facilitates the development of ankylosing spondylitis through the PER2-mediated Wnt/beta-catenin axis. Cell Death Discov. 2022 May 23;8(1):269.

46. Zhang Y, Le X, Zheng S, Zhang K, He J, Liu M, et al. MicroRNA-146a-5p-modified human umbilical cord mesenchymal stem cells enhance protection against diabetic nephropathy in rats through facilitating M2 macrophage polarization. Stem Cell Res Ther. 2022 Apr 27;13(1):171.

47. Ahmad I, Naqvi RA, Valverde A, Naqvi AR. LncRNA MALAT1/microRNA-30b axis regulates macrophage polarization and function. Front Immunol. 2023;14:1214810.

48. Li C, Li R, Hu X, Zhou G, Jiang G. Tumor-promoting mechanisms of macrophage-derived extracellular vesicles-enclosed microRNA-660 in breast cancer progression. Breast Cancer Res Treat. 2022 Apr;192(2):353-68.

49. Luo X, Meng C, Zhang Y, Du Q, Hou C, Qiang H, et al. MicroRNA-21a-5p-modified macrophage exosomes as natural nanocarriers promote bone regeneration by targeting GATA2. Regen Biomater. 2023;10:rbad075.

50. Zhao G, Yu H, Ding L, Wang W, Wang H, Hu Y, et al. microRNA-27a-3p delivered by extracellular vesicles from glioblastoma cells induces M2 macrophage polarization via the EZH1/KDM3A/CTGF axis. Cell Death Discov. 2022 May 14;8(1):260.

51. Bao L, Li X. MicroRNA-32 targeting PTEN enhances M2 macrophage polarization in the glioma microenvironment and further promotes the progression of glioma. Mol Cell Biochem. 2019 Oct;460(1-2):67-79.

52. Ning Y, Huang P, Chen G, Xiong Y, Gong Z, Wu C, et al. Atorvastatin-pretreated mesenchymal stem cell-derived extracellular vesicles promote cardiac repair after myocardial infarction via shifting macrophage polarization by targeting microRNA-139-3p/Stat1 pathway. BMC Med. 2023 Mar 16;21(1):96.

53. Zhang D, Yao X, Teng Y, Zhao T, Lin L, Li Y, et al. Adipocytes-Derived Exosomal microRNA-1224 Inhibits M2 Macrophage Polarization in Obesity-Induced Adipose Tissue Inflammation via MSI2-Mediated Wnt/beta-Catenin Axis. Mol Nutr Food Res. 2022 Sep;66(18):e2100889.

54. Peng LY, Li BB, Deng KB, Wang WG. MicroRNA-214-3p facilitates M2 macrophage polarization by targeting GSK3B. Kaohsiung J Med Sci. 2022 Apr;38(4):347-56.

55. Fan L, Xu G, Zeng X. M2 macrophage-derived extracellular vesicles augment immune evasion and development of colorectal cancer via a circRNA_CCDC66/microRNA-342-3p/metadherin axis. Cytotechnology. 2023 Aug;75(4):293-308.

56. Yin J, Zhao X, Chen X, Shen G. Emodin suppresses hepatocellular carcinoma growth by regulating macrophage polarization via microRNA-26a/transforming growth factor beta 1/protein kinase B. Bioengineered. 2022 Apr;13(4):9548-63.

57. Zhang Z-W, Wang M, Hu J-J, Xu G, Zhang Y, Zhang N. Decreased Expression of MicroRNA-107 in B Lymphocytes of Patients with Antibody-Mediated Renal Allograft Rejection. The Tohoku Journal of Experimental Medicine. 2018;246(2):87-96.

58. Jiang D, Aguiar RCT. MicroRNA-155 controls RB phosphorylation in normal and malignant B lymphocytes via the noncanonical TGF-β1/SMAD5 signaling module. Blood. 2014;123(1):86-93.

59. Xiao C, Calado Dinis P, Galler G, Thai T-H, Patterson Heide C, Wang J, et al. MiR-150 Controls B Cell Differentiation by Targeting the Transcription Factor c-Myb. Cell. 2016;165(4).

60. Wang-Renault S-F, Boudaoud S, Nocturne G, Roche E, Sigrist N, Daviaud C, et al. Deregulation of microRNA expression in purified T and B lymphocytes from patients with primary Sjögren’s syndrome. Annals of the Rheumatic Diseases. 2018;77(1):133-40.

61. Guo Q, Zhang J, Li J, Zou L, Zhang J, Xie Z, et al. Forced miR-146a expression causes autoimmune lymphoproliferative syndrome in mice via downregulation of Fas in germinal center B cells. Blood. 2013;121(24):4875-83.

62. Jiang XX, Liu Y, Li H, Gao Y, Mu R, Guo J, et al. MYSM1/miR-150/FLT3 inhibits B1a cell proliferation. Oncotarget. 2016 Oct 18;7(42):68086-96.

63. Aung LL, Mouradian MM, Dhib-Jalbut S, Balashov KE. MMP-9 expression is increased in B lymphocytes during multiple sclerosis exacerbation and is regulated by microRNA-320a. Journal of Neuroimmunology. 2015;278:185-9.

64. Ventura A, Young AG, Winslow MM, Lintault L, Meissner A, Erkeland SJ, et al. Targeted Deletion Reveals Essential and Overlapping Functions of the miR-17∼92 Family of miRNA Clusters. Cell. 2008;132(5):875-86.

65. Rao DS, O'Connell RM, Chaudhuri AA, Garcia-Flores Y, Geiger TL, Baltimore D. MicroRNA-34a Perturbs B Lymphocyte Development by Repressing the Forkhead Box Transcription Factor Foxp1. Immunity. 2010;33(1):48-59.

66. de Yébenes VG, Belver L, Pisano DG, González S, Villasante A, Croce C, et al. miR-181b negatively regulates activation-induced cytidine deaminase in B cells. The Journal of Experimental Medicine. 2008;205(10):2199-206.

67. Lai M, Gonzalez-Martin A, Cooper AB, Oda H, Jin HY, Shepherd J, et al. Regulation of B-cell development and tolerance by different members of the miR-17∼92 family microRNAs. Nature Communications. 2016;7(1).
